# Polymeric Materials for Ophthalmic Lenses: A Systematic Review on Structure, Properties, Manufacturing and Sustainability

**DOI:** 10.3390/molecules31203680

**Published:** 2026-10-14

**Authors:** Marco Calvi, Elisa Baldini, Matteo Maria Minotto, Davide Moro, Valeria Gagliardi, Domenico Santandrea, Valentina Beghetto

**Affiliations:** 1 Research and Innovation Area, Certottica S.c.r.l., Italian Institute of Certification of Optical Products, 32013 Longarone, Italy; elisa.baldini@certottica.it (E.B.); matteo.minotto@certottica.it (M.M.M.); davide.moro@certottica.it (D.M.); 2 Dipartimento di Scienze Molecolari e Nanosistemi, Università Ca’ Foscari Venezia, Via Torino 155, 30172 Venezia, Italy; valeria.gagliardi@unive.it (V.G.); domenico.santandrea@unive.it (D.S.); 3 Crossing S.r.l., Viale della Repubblica 193/b, 31100 Treviso, Italy; 4 Consorzio Interuniversitario per le Reattività Chimiche e La Catalisi (CIRCC), Via C. Ulpiani 27, 70126 Bari, Italy

**Keywords:** ophthalmic lenses, polymeric materials, structure–property relationships, surface coatings, sustainability

## Abstract

Polymeric materials have largely replaced mineral glass in ophthalmic lenses owing to their low density, impact resistance, optical performance, and manufacturing versatility. Despite their widespread use, the available literature remains fragmented, with individual studies primarily addressing specific polymer families, optical performance, manufacturing technologies, or surface coatings rather than providing an integrated overview of ophthalmic lens materials. This systematic review critically examines the relationships between chemical structure and molecular architecture of ophthalmic lens polymers and their optical, mechanical, thermal, and processing properties. The literature published between 2000 and 2026 was searched in Web of Science, Scopus, ScienceDirect, and PubMed, following PRISMA 2020 guidelines, and 91 documents were included. Properties of poly(diethylene glycol bis(allyl carbonate)) (CR-39^®^), polycarbonate, poly(methyl methacrylate), poly(urethane-urea) (Trivex^®^), and high-refractive-index poly(thiourethane) systems are reported, with particular attention to refractive index, chromatic dispersion, impact resistance, durability, processability, and coating compatibility. Surface-engineering approaches, digital manufacturing, market developments, and environmental challenges associated with production waste and end-of-life management are also evaluated. The analysis identifies persistent trade-offs between optical performance, mechanical durability, manufacturing requirements, and recyclability, highlighting the need for high-index, low-dispersion polymers, durable or repairable coating systems, and viable recovery routes for crosslinked ophthalmic materials and lens-processing waste. Emerging opportunities for material recycling, recovery, and circular economy implementation are also critically evaluated. By integrating polymer chemistry, surface engineering, manufacturing, and sustainability within a single review, this work provides a comprehensive reference for researchers and industry professionals involved in the development of next-generation ophthalmic materials.

## 1. Introduction

According to the World Health Organization, at least 2.2 billion people worldwide live with near- or distance-vision impairment, and at least 1.1 billion of these cases could be prevented in different ways, including corrective eyewear [1]. Furthermore, nearly two-thirds of people living in low-income countries with distance vision impairment lack access to appropriate corrective spectacles. This unmet need is projected to increase over the coming decades owing to population aging and the growing prevalence of refractive disorders worldwide [2].

Ophthalmic lenses constitute the primary non-invasive solution for correcting refractive errors, including myopia, hyperopia, astigmatism, and presbyopia. By functioning as dioptric media, they modify the path of incident light through refraction, allowing light rays to be properly focused on the retina and thereby restoring clear and accurate vision [3]. Recent progress in polymer synthesis has demonstrated how molecular design and functional monomer selection can be exploited to tailor mechanical strength, optical behavior, chemical resistance, and long-term durability for specialized applications. These advances have considerably expanded the range of high-performance polymeric materials available for optical and biomedical devices.

Ophthalmic lenses are recognized by European Regulation 2017/745 as Medical Devices [4]. A medical device is defined as “*any instrument, apparatus, equipment, software, implant, reagent, material or other article intended by the manufacturer to be used, alone or in combination, for one or more of the following specific medical purposes: -diagnosis, prevention, monitoring, prediction, prognosis, treatment or alleviation of disease, -diagnosis, monitoring, treatment, alleviation or compensation for an injury or disability, -investigation, replacement or modification of the anatomy or of a physiological or pathological process or state, -providing information by the in vitro examination of specimens derived from the human body, including donated blood and tissues, and which does not exert its primary intended action in or on the human body by pharmacological, immunological or metabolic means, but which may be assisted in its function by such means*”.

Ophthalmic lenses can differ in terms of weight, thickness, strength, and optical quality. The choice of the material depends on several factors, such as the type of correction required, the kind of surface coatings (for example, mirror, scratch-resistant), and the price convenience required by the end user. In general, the polymeric materials used for ophthalmic lenses differ from those employed in sunglasses because they are designed to meet distinct functional requirements. Ophthalmic lenses must accurately correct refractive errors while maintaining high optical quality, whereas sunglass lenses are primarily intended to protect the eyes from ultraviolet (UV) radiation and excessive visible light, although corrective and protective functions may also be combined in prescription sunglasses.

Plastic ophthalmic lenses have progressively replaced traditional mineral glass lenses because of their superior combination of optical, mechanical, and practical properties. In fact, compared with glass, polymeric lenses exhibit significantly lower weight, higher impact resistance, and allow the fabrication of thinner lenses, thereby improving wearer comfort and safety. For this reason, plastic lenses currently dominate the ophthalmic market, accounting for approximately 97–98% of the global market. Nevertheless, since polymeric materials generally exhibit lower surface hardness than glass, plastic ophthalmic lenses are generally treated with multiple surface coatings to enhance their optical and mechanical performances, such as improved scratch, abrasion, and impact resistance, UV protection, anti-reflective properties, anti-fog properties, and hydrophobic and oleophobic characteristics that facilitate cleaning and reduce surface contamination [3]. Consequently, coating technologies should be regarded as an integral component of modern ophthalmic polymer systems rather than as secondary post-processing treatments and will, therefore, be an integral part of this review.

From a critical analysis of reviews and research works published since 2000, it emerges that only fragmentary works have been reported regarding either historical development, general description of spectacle lens polymers, or surface treatments [3,4,5,6,7,8,9,10]. Given our expertise in materials science and environmental sustainability, this review intends to give a general overview discussing the types and characteristics of different fossil and biobased polymers for ophthalmic lens production, advantages and constraints, the relationship between polymer molecular architecture, optical and mechanical performance, manufacturing processes, coating treatments, and end-of-life management [5,7,8,9,10,11,12,13].

## 2. Methodology

This systematic review was prepared in accordance with the PRISMA 2020 statement [5,14,15]. PRISMA (Supplementary Materials, Preferred Reporting Items for Systematic Reviews and Meta-Analyses) provides a structured framework for reporting systematic reviews. It comprises a detailed checklist and a four-stage flow diagram describing how records progress through identification, screening, eligibility assessment, and final inclusion. Its application promotes transparent and methodologically rigorous reporting, facilitates reproducibility, and enables a more reliable critical appraisal of systematic reviews and meta-analyses. Owing to the broad materials science scope and the heterogeneity of the data available, including experimental studies, reviews, books, standards, technical reports, institutional documents, and manufacturer information, data were synthesized qualitatively, and no meta-analysis was performed.

The review addressed the following question: how do the chemical composition and molecular architecture of polymeric ophthalmic lens materials determine their optical and mechanical performance, processing routes, coating compatibility, durability, and end-of-life options?

The literature search covered Web of Science, Scopus, ScienceDirect, and PubMed. Reference lists of eligible publications were also searched manually to identify relevant sources not retrieved through the database search (Google Scholar). Websites and documents from the World Health Organization, European Commission, EUR-Lex, standards bodies, relevant industrial organizations, and manufacturers were also consulted. The date on which sources were last searched was 18 September 2026.

Searches used individual terms and combinations including “ophthalmic lens”, “spectacle lens”, “CR-39”, “poly(diethylene glycol bis(allyl carbonate))”, “polycarbonate”, “PMMA”, “Trivex”, “high-index lens”, “MR resin”, “urethane urea”, “thiourethane”, “ophthalmic coating”, “anti-reflective coating”, “hard coating”, “photochromic lens”, “lens manufacturing”, “freeform lens”, “ophthalmic lens recycling”, “lens waste”, “ophthalmic microplastic”, “bio-based ophthalmic polymers”, and “LCA”.

The review considered sources published from 2000 to 2026; seminal publications were retained when required to explain the historical development of materials or technologies. Eligible sources reporting information on polymer lens composition, structure–property relationships, optical or mechanical properties, processing, coatings, characterization, applications, standards, patents, market context, or end-of-life management of spectacle lenses were considered. Publications concerning contact and intraocular lenses or unrelated optical polymers were excluded. Only papers published in the English language were considered. After removal of duplicates, titles, and abstracts, papers were screened according to criteria reported above, and relevant selected documents were reviewed. No automated screening tool was used. Two authors reviewed the extracted data from the studies. The information was organized thematically according to polymer family and functional topic and was compared to identify relationships between material structure, performance, processability, coating compatibility, durability, and sustainability. The study selection process is summarized in Figure 1, and 98 reference documents were selected.

## 3. Human Vision and the Optical Function of Ophthalmic Lenses

Vision results from the coordinated action of the optical components of the eye, which collect, refract, and focus incoming light onto the retina, where photoreceptor cells convert the optical image into electrical signals that are transmitted through the optic nerve to the visual cortex of the brain for image processing [16]. The eye functions as a highly sophisticated optical system in which the cornea and crystalline lens provide most of the refractive power required to produce a sharp retinal image. Under normal conditions (emmetropia), parallel light rays originating from distant objects are focused precisely on the retinal plane, enabling clear vision.

Approximately two-thirds of the total refractive power of the eye (about 43 dioptres) is provided by the cornea owing to the large refractive index difference between air and the corneal tissue, whereas the crystalline lens contributes approximately 17–20 dioptres while simultaneously providing accommodation. Accommodation is achieved through contraction and relaxation of the ciliary muscle, which modifies the curvature of the crystalline lens and, therefore, its optical power, allowing the eye to maintain focus on objects located at different distances. This dynamic adjustment gradually decreases with age because of lens stiffening, eventually leading to presbyopia.

Clear vision depends on the precise convergence of light rays onto the retina. Whenever the optical power of the eye does not match its axial length or when the refractive surfaces are irregular, the focal point is displaced either in front of or behind the retina, producing refractive errors [17]. Myopia occurs when the optical power is excessive or the eyeball is too long, causing distant objects to focus in front of the retina. Conversely, hyperopia results from insufficient optical power or a shorter axial length, shifting the focal point behind the retina. Astigmatism arises from non-uniform curvature of the cornea or crystalline lens, generating different focal lengths along different meridians (Figure 2).

Instead, presbyopia results from the age-related reduction in accommodative ability of the crystalline lens. Together, these conditions represent the most common causes of correctable visual impairment worldwide.

Ophthalmic lenses compensate for these refractive errors by introducing a precisely calculated optical power that redirects incoming light before it enters the eye. By appropriately converging or diverging light rays, spectacle lenses restore the focal point to the retinal plane, allowing a sharp image to be formed without altering the anatomical structure of the eye itself. Concave (negative) lenses are used to correct myopia by diverging incident light, whereas convex (positive) lenses converge light to compensate for hyperopia and presbyopia. Cylindrical or toric lens geometries provide different optical powers along specific meridians to correct astigmatism, while multifocal and progressive addition lenses combine multiple optical powers within a single lens to provide clear vision at far, intermediate, and near distances.

Although the primary function of ophthalmic lenses is optical correction, modern spectacle lenses have evolved into highly engineered devices that integrate advanced polymeric materials, sophisticated surface treatments, and precision optical designs [18]. Contemporary lenses are expected not only to provide accurate refractive correction but also to exhibit high transparency, low weight, excellent impact and scratch resistance, UV protection, anti-reflective performance, and long-term durability [18]. Consequently, the development of polymeric materials for ophthalmic lens applications requires the simultaneous optimization of optical, mechanical, and physicochemical properties, making polymer chemistry a fundamental aspect of modern ophthalmic lens technology.

## 4. Polymeric Materials for Ophthalmic Lenses

The development of ophthalmic lenses has been closely associated with advances in polymer science, leading to the introduction of materials with progressively improved optical, mechanical, and physicochemical properties. Today, a wide range of polymeric materials are available for ophthalmic lens applications, each offering a specific balance between refractive index (RI), optical quality, density, impact resistance, processability, and cost. Consequently, material selection strongly depends on the final application and performance required.

### 4.1. Structure–Property Relationships in Ophthalmic Polymers

Ophthalmic lens materials can be broadly classified, according to their molecular architecture and processing technique, into thermoplastic and thermosetting polymers. Thermoplastic materials, such as polycarbonates (PCs) and polymethacrylates, consist of linear or slightly branched macromolecules that soften upon heating and can be repeatedly processed by melt techniques. In contrast, thermosetting materials, including poly(diethylene glycol bis(allyl carbonate)) resins (CR-39^®^), poly(urethane-urea) (for example Trivex^®^), and modern high-index thiourethane resins, form permanently crosslinked three-dimensional networks during curing, providing superior dimensional stability and optical homogeneity but preventing conventional melt reprocessing. The chemical structure of the main monomers employed for the synthesis of these polymers is schematically reported in Figure 3 [19,20,21,22,23].

Their performance is governed not only by chemical composition but also by molecular architecture, nature of the polymer backbone, degree of crosslinking, and mobility of the polymer chains. These structural features collectively determine the optical, mechanical, and thermal properties required for ophthalmic applications, such as RI, chromatic dispersion (Abbe number), impact resistance, dimensional stability, and processability, which must be verified according to standard tests [3,24,25,26,27,28,29,30]. The RI and the Abbe number are among the most important optical properties governing ophthalmic lens performance, determining the curvature and thickness required to obtain a given RI, and quantifying the optical dispersion of a material (Abbe number) [31]. In particular, this latter determines the extent to which RI varies across different wavelengths of visible light, so that materials with high Abbe numbers exhibit lower dispersion and chromatic aberration, whereas materials with low Abbe numbers may generate visible colour fringes, particularly in the peripheral region of the lenses [3,25].

The RI of a polymer is governed by the electronic polarizability of its constituents, namely, the ease with which electron clouds are distorted by the oscillating electric field of light [24,25,32]. Consequently, molecular structures containing highly polarizable groups, such as aromatic rings, halogenated moieties, or sulfur atoms, generally exhibit higher refractive indices as a consequence of stronger interactions with the incident electromagnetic radiation. This relationship can be expressed quantitatively through the Lorentz–Lorenz equation:

(1) $$\frac{n^{2}-1}{n^{2}+2}=\frac{R_{m}}{V_{m}}$$

where *n* is the refractive index, *R*_m_ is the molar refraction, which reflects the electronic polarizability of the constituent atoms and functional groups, and *V*_m_ is the molar volume. Accordingly, the RI of a polymer can be increased either by introducing groups with high molar refraction or by decreasing molar volume through more efficient molecular packing. In ophthalmic materials, however, these strategies must be balanced against their possible effects on chromatic dispersion, density, coloration, and chain mobility. In particular, highly polarizable π-electron systems of aromatic rings contribute to improving RI and at the same time restricting rotational mobility along the polymer backbone, contributing to the dimensional stability of the material. Nevertheless, in aromatic-containing polymers, such as poly(bisphenol A carbonate) (BPA-PC), the final optical and thermomechanical properties also depend on carbonate linkages, molecular weight, chain packing, free volume, and processing-induced molecular orientation [25,32]. Thus, increasing aromatic content does not necessarily improve overall optical quality, and the spatial arrangement of aromatic groups and their effect on chain orientation must also be considered.

A very effective strategy to increase the RI of optical polymers is the incorporation of sulfur atoms, which are considerably more polarizable than oxygen or carbon atoms. For this reason, sulphide, thiocarbonyl, and thiocarbamate are functionalities widely present in high-RI optical lenses [33]. Recently devised poly(thiourethane) lens materials combine sulfur-containing monomers with multifunctional isocyanates, reaching RI values substantially higher than those of CR-39^®^ or PMMA [34,35]. However, increasing polarizability frequently enhances the wavelength dependence of RI, and high-RI materials containing sulfur atoms generally exhibit lower Abbe numbers [32]. RI and chromatic dispersion must, therefore, be balanced through the overall monomer architecture rather than by simply maximizing sulfur concentration.

Crosslinking density has a similarly important effect on lens performance. Thermoset materials such as CR-39^®^, Trivex^®^, and poly(thiourethane) form permanent three-dimensional networks during curing. Crosslinking improves dimensional stability, creep, and solvent resistance, together with optical geometry stability during use. Nevertheless, very high crosslinking density increases materials stiffness and brittleness, together with optical distortion during processing [25]. Consequently, the final properties of thermoset lenses depend not only on monomer chemical structure and nominal crosslink density but also on network homogeneity and curing conditions. Crosslink density cannot, however, be related to impact resistance through a single universal relationship across the different ophthalmic polymer families. The measured response also depends on segment flexibility, glass transition temperature, residual stresses, specimen geometry, conditioning, and test procedure. Quantitative values obtained from different formulations or non-equivalent impact tests should, therefore, not be directly compared.

Molecular architecture also directly determines processability and end-of-life options. Linear thermoplastic polymers, such as BPA-PC and PMMA, can be softened and reshaped above their glass transition temperatures and can be melt-reprocessed. However, repeated thermal processing may cause molecular weight reduction, yellowing, or changes in optical properties, and the presence of coatings and additives complicates the recovery of optical-grade material. In contrast, the covalently crosslinked networks of CR-39^®^, Trivex^®^, and poly(thiourethane) cannot be remelted after curing. Their end-of-life management consequently requires mechanical size reduction, use as fillers, chemical network cleavage, or other thermoset-valorization strategies rather than conventional remelting [36].

Within this panorama, it is evident that no single molecular architecture simultaneously maximizes all properties required for ophthalmic lenses. Increasing aromaticity, sulfur content, or molecular packing can increase RI and reduce lens thickness but may also increase dispersion, birefringence, density, or coloration. Increasing crosslink density improves chemical and dimensional stability but limits ductility and recyclability, whereas greater chain mobility improves impact energy dissipation but can reduce surface hardness and increase sensitivity to deformation. The selection and design of ophthalmic polymers, therefore, require a compromise among RI, chromatic dispersion, transparency, impact resistance, surface hardness, density, processability, and end-of-life management, as summarized in Table 1.

The suitability of a polymer or coating system is verified on the finished lens using standardized requirements and test methods. ISO 14889 [28] specifies the fundamental requirements applicable to uncut finished spectacle lenses, whereas the ISO 8980 series provides more specific requirements and test methods for optical power, geometrical characteristics, transmittance, and surface performance [26,27]. In particular, ISO 8980-4 [26] addresses the properties of anti-reflective and hydrophobic coatings, while ISO 8980-5 [27] defines minimum requirements for lens surfaces claimed to be abrasion-resistant. ANSI Z80.1 provides corresponding recommendations and tolerances for prescription ophthalmic lenses in the United States, particularly for edged or assembled lenses, while also applying relevant optical and physical specifications to uncut lenses [29]. Within this general framework, the specific properties of main polymer families employed for ophthalmic lenses such as CR-39^®^, PCs, PMMA, poly(urethane-urea) (Trivex^®^), and poly(thiourethane) (MR^TM^) are described in the sections below.

### 4.2. Allyl Carbonate Polymers (CR-39^®^)

Poly(diethylene glycol bis(allyl carbonate)), commercially known as CR-39^®^, is a highly crosslinked thermoset polymer introduced during the 1940s by PPG Industries (Pittsburgh Plate Glass Company, Pittsburgh, PA, USA) to develop transparent polymeric resins for optical applications (Figure 3) [5,6]. The name CR-39^®^ originates from the fact that it was the thirty-ninth formulation developed during the “Columbia Resins” research program conducted by PPG Industries.

CR-39^®^ is polymerized through radical curing of allyl double bonds, producing a densely crosslinked three-dimensional network which is responsible for its excellent optical clarity, chemical resistance, optical homogeneity, and dimensional stability. With one of the highest Abbe numbers available on the market, CR-39^®^ remains the reference material for conventional ophthalmic lenses (Table 1) [5]. However, its relatively low RI (approximately 1.50, Table 1) results in thicker lenses for high prescriptions, while restricted network mobility limits impact energy dissipation compared with tougher materials, such as BPA-PC or Trivex^®^ [5]. Furthermore, the material is susceptible to surface scratching, and CR-39^®^ lenses need to be protected by hard coatings [5,7]. As far as its end of life is concerned, the allyl carbonate network cannot be conventionally reprocessed by melting and, therefore, its recyclability is rather problematic.

### 4.3. Polycarbonates

Polycarbonates (PCs) are thermoplastic polymers containing carbonate groups in their molecular backbone and can be classified into aromatic and aliphatic polycarbonates according to their chemical structure. The aromatic poly(bisphenol A carbonate) (BPA-PC) is by far the most widely used PC for ophthalmic lenses because of its excellent optical transparency, impact resistance, dimensional stability, and processability (Figure 3) [12].

Introduced on the market in 1957, BPA-PC rapidly became the material of choice for safety eyewear, sport glasses, and children’s spectacles because of its wearability and exceptional impact resistance [6,37]. Its aromatic structure provides stiffness and dimensional stability, together with sufficient mobility and free volume available for local molecular rearrangement and plastic deformation under impact loading. These mechanisms enable BPA-PC to dissipate substantial energy before fracture, accounting for its exceptional impact resistance, good dimensional stability, resistance to high temperatures, and weathering [12]. Compared with CR-39^®^, BPA-PC has a lower density and a higher RI (1.50 and 1.59, respectively, Table 1), enabling the production of thinner and lighter lenses while maintaining excellent mechanical performance. An interesting feature of BPA-PC, compared to other high-RI materials, is its intrinsic ability to absorb UV radiation, avoiding the use of UV stabilizers and other additives to maintain long-term optical performance [5,38,39]. The main disadvantage of BPA-PC is the relatively low Abbe number (~31; Table 1), resulting in greater chromatic dispersion and potentially noticeable peripheral chromatic aberrations in high prescription lenses [6]. Moreover, lower surface hardness makes BPA-PC susceptible to scratching, while molecular orientation and residual stresses introduced during injection moulding can cause birefringence. These limitations explain why BPA-PC lenses generally require hard coatings and carefully controlled processing conditions [39].

### 4.4. Polymethacrylates

Polymethacrylates were developed in the 1930s after World War I [5]. Polymethacrylates include materials like PMMA, poly(methylacrylate), and poly(hydroxyethylmethacrylate), with the former being the most commonly used polymer for optical applications [34]. PMMA was one of the earliest polymeric materials developed for optical applications because of its exceptional transparency, low density, and favorable optical properties. PMMA is an amorphous thermoplastic polymer, where the bulky α-methyl and ester substituents restrict chain rotation and produce a relatively rigid, glassy material. This structure provides high visible light transparency and a high Abbe number (Table 1). Its relatively low polarizability also accounts for an RI of approximately 1.49 (Table 1), which limits its use for thin high-prescription lenses [32,34]. Additionally, the limited capacity of PMMA chains to undergo extensive plastic deformation promotes brittle fracture and gives considerably lower impact resistance than BPA-PC. Since ophthalmic lenses are classified as medical devices in many countries, impact resistance represents a fundamental safety requirement [29]. Consequently, following the commercial introduction of CR-39^®^, PCs, and later Trivex^®^, PMMA was progressively replaced by these tougher materials. Today, its use in ophthalmic applications is largely limited to non-prescription reading glasses, demonstration lenses, and other low-demand optical products.

### 4.5. Poly(Urethane-Urea) (Trivex^®^)

Trivex^®^ lenses were launched under different commercial names like Phoenix/Hilux/Hilux-PNX (Hoya, Tokyo, Japan), Trilogy (Younger, Torrance, CA, USA), and Trexa/NXT/Aris (Essilor, Charenton-le-Pont, France) [13]. First produced by PPG Industries in 2001, Trivex^®^ is a highly crosslinked thermoset urethane urea-based polymer prepared by casting starting from a poly(urethane-urea) prepolymer with an aromatic diamine curing agent. Urethane and urea groups establish strong intermolecular interactions, including hydrogen bonding, which contribute to cohesive strength and chemical resistance against solvents. At the same time, flexible segments between the crosslinking points allow greater energy dissipation than in more rigid thermoset networks. This combination accounts for its low density, high impact resistance, favorable mechanical performance, high transparency, and dimensional stability, making it particularly suitable for rimless frames [5,13]. Its intermediate RI of approximately 1.53 and Abbe number of 43–45 (Table 1) offer an excellent balance between optical performance and comfort [8]. These characteristics have promoted the use of Trivex^®^ in contexts where both mechanical and chemical resistance are needed, such as, for example, in industrial or medical use.

### 4.6. Polythiourethanes (MR^TM^)

Polythiourethanes are high refractive index polymers, commonly known as a high-end category lenses, i.e., premium optical products that deliver superior visual clarity, material quality, and advanced manufacturing processes [5,40]. Between 1987 and 2000, Mitsui produced different thiourethane-based polymers sold under the acronyms MR6- MR10 and MR-174, with a high RI ranging from 1.67 to 1.73 [34]. As reported above, the RI of poly(thiourethane) depends on the presence of sulfur atoms, which exhibit much higher electronic polarizability than oxygen or carbon atoms [32]. Therefore, incorporation of sulfur-containing groups (such as, for example, thioethers, thiourethane, thiiranes) significantly increases RI without excessively increasing density. This principle underlies the design of modern MR^TM^ materials developed by Mitsui Chemicals Inc. (Tokyo, Japan).

Polythiourethane lens materials combine highly polarizable sulfur-containing groups with a crosslinked network formed through reactions between multifunctional thiols and isocyanates. Sulfur increases RI, while the thiourethane groups contribute to intermolecular cohesion and chemical resistance. Variation of thiol functionality, sulfur content, isocyanate structure, and crosslinking density enables tailoring the RI over a relatively wide range. Nevertheless, the higher RI is generally accompanied by lower Abbe numbers and greater density, while excessive network rigidity may reduce impact resistance. Precise control of monomer stoichiometry and curing conditions is, therefore, required to avoid network heterogeneity and internal stresses that may compromise optical and mechanical performance. Within high-RI polymers, the MR^TM^ series represents a true turning point in modern optics, allowing the production of lighter and thinner lenses than glass or plastic counterparts, making them the ideal choice for people with high prescriptions. These polymers represent not only a technical advance but a social transformation in the way people with high ametropia relate to their spectacles [8].

## 5. Lens Coating and Deposition Technologies

Polymeric materials, generally used for lens production, exhibit lower surface hardness than mineral glass and, therefore, multiple surface treatments and coatings have been developed to enhance their durability, anti-reflective properties, anti-fog properties, UV protection, resistance to scratches and abrasion, and glare reduction, in addition to hydrophobic and oleophobic properties that facilitate the cleaning process [3,5,41,42]. These coatings comprise a combination of organic, inorganic, and hybrid materials that are sequentially deposited onto the polymer substrate to obtain complementary properties, as described below and depicted in Figure 4.

### 5.1. Hard Coatings

The first coating applied to polymeric ophthalmic lenses is generally a hard coating to improve scratch and abrasion resistance while providing a suitable substrate for further coating deposition. Hard coatings are typically based on highly crosslinked polysiloxane or organically modified silicate networks obtained through the hydrolysis and condensation of alkoxysilanes via sol–gel processes. Commercial formulations frequently contain colloidal silica nanoparticles dispersed within the siloxane matrix, producing transparent organic–inorganic nanocomposites with significantly increased hardness while maintaining excellent optical transparency. In addition to improving mechanical durability, these coatings also enhance adhesion between the polymer lens and the inorganic anti-reflective layers deposited afterwards [43].

The hardening treatment is carried out using three different techniques (sublimation, lacquering, and nanocomposite deposition), after which the lenses are placed in an oven to dry the deposited layer of material [44]. The sublimation process involves placing the lens under vacuum inside a glass bell jar where the hardening material transitions from solid to gaseous state, and particles released are deposited onto the lens. This technique produces very thin layers, resulting in modest levels of protection [45]. Alternatively, immersion or centrifugation lacquering may be carried out using silicone-based paints (lacquers), which provide high resistance with thin coatings. Nanocomposite paints are new-generation silicone lacquers containing dispersed silicon microparticles and enabling improvement of surface hardness up to 30% compared to standard lacquers [41].

### 5.2. Anti-Reflective Coatings

Anti-reflective coatings are dielectric thin-film systems designed to reduce reflection losses through destructive interference between light waves reflected at adjacent interfaces [46]. According to the Fresnel relationship, the fraction of light reflected at the air–lens interface increases with the refractive index difference between the two media. Anti-reflective treatments are, therefore, particularly important for high-RI polymer lenses. In the simplest design, a coating with an optical thickness corresponding to approximately one quarter of the selected wavelength reduces reflection over a relatively narrow spectral range. Modern broadband coatings instead comprise several alternating high- and low-RI layers. The thickness and sequence of these layers are optimized using characteristic matrix calculations, which predict the reflectance of the complete coating system as a function of wavelength and angle of incidence.

SiO_2_ and MgF_2_ are representative low-index materials, whereas TiO_2_, ZrO_2_, Ta_2_O_5_, Nb_2_O_5_, and HfO_2_ provide a higher RI. Nevertheless, selection of a particular multilayer architecture cannot be based on RI alone, since absorption, adhesion to the hard coat, residual stress, porosity, resistance to humidity and abrasion, deposition temperature, and compatibility with the polymer substrate must also be considered [47,48]. Unlike predominantly organic or organic–inorganic hard coatings, anti-reflective layers are generally inorganic and are deposited by physical vapor deposition techniques, including vacuum evaporation, ion-assisted deposition, and magnetron sputtering, or by plasma-assisted processes [49]. During these processes, the coating materials are evaporated or sputtered under vacuum and subsequently condensed onto the lens surface. Ion assistance may additionally be employed to control film density, adhesion, microstructure, and residual stress [47,48,50].

### 5.3. Hydrophobic and Oleophobic Top Coatings

The outermost layer of premium ophthalmic lenses is generally an ultrathin hydrophobic and oleophobic coating designed to minimize water, oils, and particulate adhesion while facilitating lens cleaning. These coatings are typically based on fluorinated organosilane or fluoropolymer derivatives capable of forming covalently bonded self-assembled monolayers through siloxane (Si–O–Si) linkages with the underlying oxide surface. The fluorinated alkyl chains orient towards the air interface, producing very low surface energy and consequently reducing wetting by both aqueous and oily liquids [51].

### 5.4. Functional Coatings

Additional functional coatings may be incorporated depending on the intended application of the ophthalmic lens. Photochromic coatings generally contain organic photochromic molecules, including naphthopyrans, spirooxazines, or spiropyrans, which undergo molecular rearrangements upon UV irradiation, producing a reversible increase in optical absorbance [52]. Mirror coatings are typically formed by thin metallic films or dielectric multilayers that selectively reflect part of the incident light while maintaining optical transparency [53].

Anti-fog coatings generally rely on hydrophilic polymeric networks or organic–inorganic hybrid coatings capable of spreading condensed water into a uniform transparent film rather than discrete droplets, thereby preserving optical clarity under conditions of high humidity or rapid temperature changes [5]. Sometimes, to combine anti-fogging and UV protection, photochromic coatings are used, which make ophthalmic lenses change to a dark tint in the sunlight and clear indoors thanks to a response to activation and deactivation by UV radiation [54]. Lastly, the blue block coating, which has become popular in recent years, has the aim to filter the visible blue light (400 to 500 nm wavelength), which can cause eye strain from digital devices [54,55,56,57]. A summary of the main coatings employed, their characteristics, and deposition methods is reported in Figure 4 and Table 2.

In conclusion, coating technologies described above provide complementary rather than interchangeable functions. Polysiloxane and organic–inorganic hybrid hard coats primarily improve the scratch and abrasion resistance of polymeric lenses and provide a mechanically compatible intermediate layer for the deposition of inorganic anti-reflective coatings. Their performance depends on coating composition and thickness, nanoparticle dispersion, curing conditions, and the mechanical mismatch between the coating and polymer substrate [7,58]. Multilayer dielectric anti-reflective coatings reduce surface reflectance and consequently increase visible light transmission and reduce glare. Their durability depends on interfacial adhesion, residual stress, layer structure and porosity, and resistance to humidity, temperature variations, radiation, and mechanical rubbing [58]. Hydrophobic and oleophobic top coats are ultrathin surface treatments whose principal function is to reduce surface energy, water and oil adhesion, and surface contamination, thereby facilitating cleaning rather than providing substantial mechanical reinforcement. Because optical performance and durability depend on the complete multilayer architecture and on the test conditions employed, coating systems should be evaluated on finished coated lenses using standardized measurements of reflectance, water repellence, adhesion, environmental resistance, and rubbing durability [7,58].

## 6. Ophthalmic Lens Manufacturing and Digital Surfacing

The manufacture of ophthalmic lenses is a highly technological and automated process, which is carried out to convert raw materials into semi-finished and finished lenses [59]. Preliminary manufacturing stages foresee casting or injection moulding, depending on whether the selected polymer is a thermoset or a thermoplastic. Lenses may be supplied as finished single-vision lenses or as semi-finished blanks having a defined front surface with a specific curve and thickness, and an unfinished back surface, which is customized according to wearer-specific prescription [60,61]. Conventional surfacing produces relatively simple spherical or toric surfaces, whereas digital freeform surfacing uses computer-controlled generators to produce different surface geometries [62]. This approach allows the lens surface to incorporate the required characteristics (sphere, cylinder, axis, prism, power) and facilitates the production of customized single-vision and progressive lenses. Freeform design can also be used to control the distribution of unwanted astigmatism and other optical aberrations across the lens surface [63].

Then, the lenses are attached to a block piece using a special low-melting-point alloy or UV adhesive, which holds the lens inside the grinding machines and produces the final three-dimensional curvature [61]. Finally, lenses undergo several surface treatments depending on the final properties required. At this stage, the lens is optically complete. In the finishing laboratory, lenses are shaped to the exact size of the frame and cut down to size (dry or wet edging) [50].

## 7. Economic and Environmental Impacts

The global ophthalmic lens market was valued at approximately USD 38.1 billion in 2024 and is projected to reach USD 55.92 billion by 2033, corresponding to a compound annual growth rate of 4.36% over the forecast period [64]. At the global level, companies recognized as key players of the ophthalmic lens market are Carl Zeiss AG, Essilor Luxottica, Guangzhou Hechang Optical Co., Horizons Optical, HOYA Vision Care, Indizen Optical Technologies, Luxexcel Group B.V., Mason Lenses, Mitsui Chemicals Inc., Nikon Essilor Co., Ltd., Phenix Optics Co., Ltd., Rodenstock GmBH, Schneider GmbH and Co. KG, Seiko Optical Products Co., Ltd., Shamir Ltd., Shanghai Conant Optical Co., Togusa Inc., Tokai Optical Co., Ltd., Vision Ease, and Younger Optics [65].

The defining feature of the optical products supply chain is the significant structural and value difference between the industrial system and the optical retailer distribution system. The latter is the endpoint of a complex system of companies that supply lenses, consisting of the frame and sunglasses sector, the ophthalmic industry, which produces lenses and all systems that use optical reading instruments. Finally, the pharmaceutical sector produces contact lenses, both for everyday use and for use in refractive surgery and as therapeutic instruments. The business-to-consumer market can be divided into the popular market, usually selling low- and medium-index lenses (more affordable prices), and the premium market, for high-RI lenses (higher prices). Murmura et al. reported that ophthalmic lenses have a price per unit of 79.52 euros compared to 25.64 euros for a pair of sunglasses [63].

From an environmental perspective, effective end-of-life management strategies for ophthalmic lens waste remain largely undeveloped. According to industry estimates, approximately 1.3–1.4 billion corrective ophthalmic lenses are manufactured worldwide each year, corresponding to about 650–700 million pairs of spectacles [66]. Once discarded, ophthalmic lenses are generally disposed of through landfilling or incineration. To make matters worse, most polymers employed to produce ophthalmic lenses are poorly recyclable thermosets, and only a minor part is made of potentially recyclable materials (PCs and PMMA). Additionally, the presence of considerable amounts of coatings further complicates recycling options.

Additionally, during lens edging, grinding, and polishing, substantial quantities of polymeric material are removed, generating residues ranging from millimeter-sized fragments to micro- and nano-plastics that are conveyed into process wastewater [36,67]. Since these operations typically remove approximately 50–70 wt.% of the original lens, more than 5800 tons of polymeric particles have been estimated to be generated annually worldwide [36,68]. Furthermore, the study by Lee et al. reported that 1 L of wastewater generated during lens grinding contained approximately 1380–62,539 microplastic particles and 0.0136–0.0324 mg of nanoplastics [68]. Environmental concern is further confirmed by recent papers reporting that in 2020 the UK optometry sector produced a total annual carbon footprint of 135,573 kgCO_2eq_ [69,70].

To limit microplastic release into the environment, modern edging systems increasingly incorporate closed-loop water recirculation combined with mechanical filtration, enabling the efficient separation of suspended particles prior to water reuse or discharge [71,72,73,74]. Nevertheless, recovered microplastic residues are still predominantly managed through landfilling or incineration, despite these waste streams potentially representing valuable secondary raw material for other industrial manufacturing sectors. A first example has been reported by Encarnação et al. for the recycling of thermoset microplastics, generated during lens grinding, as fillers in cement composites [36]. Alternatively, to reduce the environmental burden of ophthalmic lens production, recent research is gradually shifting from simply maximizing microplastic removal towards integrated management strategies that combine particle capture with biological degradation, phytoremediation, catalytic pyrolysis, and gasification, with the common objective of transforming plastic waste into valuable secondary resources rather than treating it solely as a residue requiring disposal [75,76,77].

## 8. Future Outlook: Emerging Materials and Manufacturing Opportunities

Recent emerging research is exploring bio-based feedstocks, recyclable or chemically cleavable high-refractive-index polymers, and additive manufacturing as possible routes towards more sustainable and customized optical components, with potential future applications for ophthalmic lenses [5,25,36,78,79,80,81,82].

For example, Fang et al. reported the synthesis of high-refractive-index polymers from phosphorus-containing derivatives of a bio-based aromatic aldehyde (4-hydroxybenzaldehyde) by a thiol–ene reaction [83]. These polymers exhibited refractive indices of 1.63–1.72 and Abbe numbers between 15.9 and 16.3, high-temperature stability, and transmittance, demonstrating how a renewable aromatic precursor can potentially be used for the preparation of high-quality ophthalmic lenses. More recently, bio-based phosphate-containing aromatic monomers bearing methoxy, allyl, or vinyl substituents were polymerized with thiophenols or mercaptans through photoclick thiol–ene reactions, producing materials with an RI of 1.63–1.70 and Abbe numbers up to 38.5 [84,85,86,87]. Data reported evidence that substituent selection affects both refractive index and chromatic dispersion. In particular, vinyl-substituted monomers provided a more favorable combination of RI and Abbe number than their allyl-containing analogs [84]. These findings confirm that maximization of sulfur or aromatic content alone is insufficient and that the entire monomer architecture must be designed to balance refractive index, dispersion, transparency, and processability.

Bio-based polycarbonates have also been studied, providing a complementary molecular design strategy [88,89,90]. Isosorbide is a renewable and conformationally rigid bicyclic diol that can improve polymer stiffness and thermal resistance. However, the alicyclic and ether-rich structure of isosorbide-based polycarbonates generally provides only moderate RI and may be associated with water absorption and orientation-dependent birefringence. Copolymerization with aromatic or fluorene-based anthraquinone monomers has proved to increase electronic polarizability, improve hydrophobicity, and regulate birefringence of the bio PC [62].

Another possible approach for the preparation of bio-based polymers for ophthalmic applications may rely on the use of renewable polysaccharides. Nevertheless, the available literature does not provide quantitative refractive index, Abbe number, or long-term durability data to demonstrate their suitability as bulk high-index spectacle lens substrates [82]. Their high concentration of hydrophilic hydroxyl or amino groups may also increase sensitivity to moisture unless appropriate chemical modification, crosslinking, or protective coating strategies are employed. At present, these materials, therefore, appear more realistic as components of functional coatings, blends, or hybrid systems than as direct replacements for commercial high-index thermoset lens matrices.

Despite these promising results, bio-based origin should not be automatically associated with biodegradability, recyclability, or lower environmental impact. Sustainability depends not only on the renewable carbon content but also on feedstock production, monomer synthesis and purification, solvent and energy consumption, processing, service lifetime, and end-of-life management [78,91]. Moreover, aromatic and sulfur-containing structures introduced to increase RI may also affect color, chromatic dispersion, viscosity, curing behavior, density, and long-term photostability [83,85]. Ophthalmic lenses must retain optical transparency, dimensional stability, and coating adhesion during prolonged exposure to humidity, heat, UV radiation, and repeated cleaning. Thus, to reduce fossil-based polymer consumption and improve life-cycle impacts while retaining the required optical and mechanical performance, much work still has to be done [25].

Another promising frontier is the introduction of chemically cleavable or dynamic covalent linkages into crosslinked optical networks. Such architectures could permit repair, reshaping, or chemical recovery under controlled conditions while retaining the dimensional stability required during use. Recent research on dynamic thiourethane networks has demonstrated the possibility of combining high refractive index, optical transparency, and chemical recyclability. However, the exchange reactions must remain effectively inactive under normal service conditions, including exposure to heat, humidity, UV radiation, and repeated cleaning. Their applicability to commercial ophthalmic lenses, therefore, requires further assessment of optical stability, durability, processing conditions, and compatibility with multilayer coatings. As far as additive manufacturing is concerned, it allows us to produce customized optical geometries, while potentially reducing the quantity of material removed during conventional lens edging [92,93]. It can also facilitate the production of complex geometries that are difficult to obtain using conventional manufacturing methods [78,94,95]. Nevertheless, printing alone is insufficient to produce finished optical-quality surfaces. Additive manufacturing must be combined with suitable post-curing, surface planarization, precision polishing or smoothing, and optical-coating deposition to reduce layer-induced roughness, light scattering, polymerization shrinkage, and refractive index heterogeneity. For this reason, optical-grade 3D-printed lenses continue to face challenges related to dimensional accuracy, surface roughness, layer-induced scattering, refractive index homogeneity, shrinkage during polymerization and post-curing, production rate, and compatibility with subsequent polishing and coating processes [94,95,96]. Scalability, reproducibility, long-term safety, and regulatory validation would constitute additional requirements before such components could be marketed as prescription spectacle lenses [91]. These technologies should, therefore, be regarded as promising research and specialized manufacturing approaches rather than mature replacements for conventional cast or injection-moulded ophthalmic lenses.

## 9. Conclusions

Polymeric materials have revolutionized the ophthalmic lens industry, progressively replacing mineral glass due to their superior optical performance and manufacturing versatility. Currently, a wide range of materials, including CR-39^®^, polycarbonates, PMMA, Trivex^®^, and high-refractive-index poly(thiourethane) resins, are available, each providing a different balance between RI, chromatic dispersion, mechanical performance, processability, durability, and cost. These differences originate primarily from the molecular architecture of the polymers, including chain structure, crosslinking density, and the incorporation of highly polarizable functional groups, such as aromatic and sulfur-containing moieties.

Beyond the polymer substrate itself, modern ophthalmic lenses are highly engineered multi-material systems in which advanced surface coatings play a fundamental role in improving scratch resistance, optical transmission, UV protection, anti-reflective behavior, hydrophobicity, and durability. Continuous developments in coating technologies, together with advances in freeform manufacturing and precision processing, have significantly expanded the performance and customization of ophthalmic lenses while supporting the growing demand generated by the increasing occurrence of refractive disorders worldwide.

Despite these technological advances, important challenges remain. From a performance point of view, the increasing need for high refractive index lenses frequently entails compromises in chromatic dispersion, density, and coloration. Additionally, assembly of chemically dissimilar polymers and inorganic coatings requires high adhesion and optical uniformity during thermal cycling, cleaning, abrasion, and long-term environmental exposure. From an environmental perspective, increasing use of permanently crosslinked thermosetting polymers, although beneficial for optical quality and dimensional stability, considerably complicates end-of-life management since these materials cannot be melt reprocessed. At the same time, lens manufacturing and finishing generate significant quantities of polymeric waste and microplastics, while recycling practices within the optical sector remain limited. Consequently, the development of circular strategies for ophthalmic materials, including mechanical recycling, material valorisation, design for recyclability, and the development of more sustainable polymer and biopolymer formulations, is becoming increasingly important to reduce the environmental footprint of the sector.

Overall, this review provides an integrated and critical framework connecting the chemistry and molecular architecture of ophthalmic polymers with their optical and mechanical performance, manufacturing requirements, coating compatibility, durability, and end-of-life behavior. Furthermore, it highlights the need for future research aiming to combine high optical performance with circular design. The main challenges include high-index, low-dispersion polymer design, durable or repairable coating stacks, reliable identification and separation of discarded lenses, and chemical or thermochemical valorization routes for crosslinked networks. Bio-based feedstocks and additive manufacturing may offer additional opportunities, but their optical homogeneity, durability, safety, and life-cycle benefits remain to be demonstrated. Finally, recycling routes of discarded microplastics produced during lens edging and grinding would highly improve the environmental sustainability of ophthalmic lens production, but still largely remain to be investigated.

## Figures and Tables

**Figure 1:**
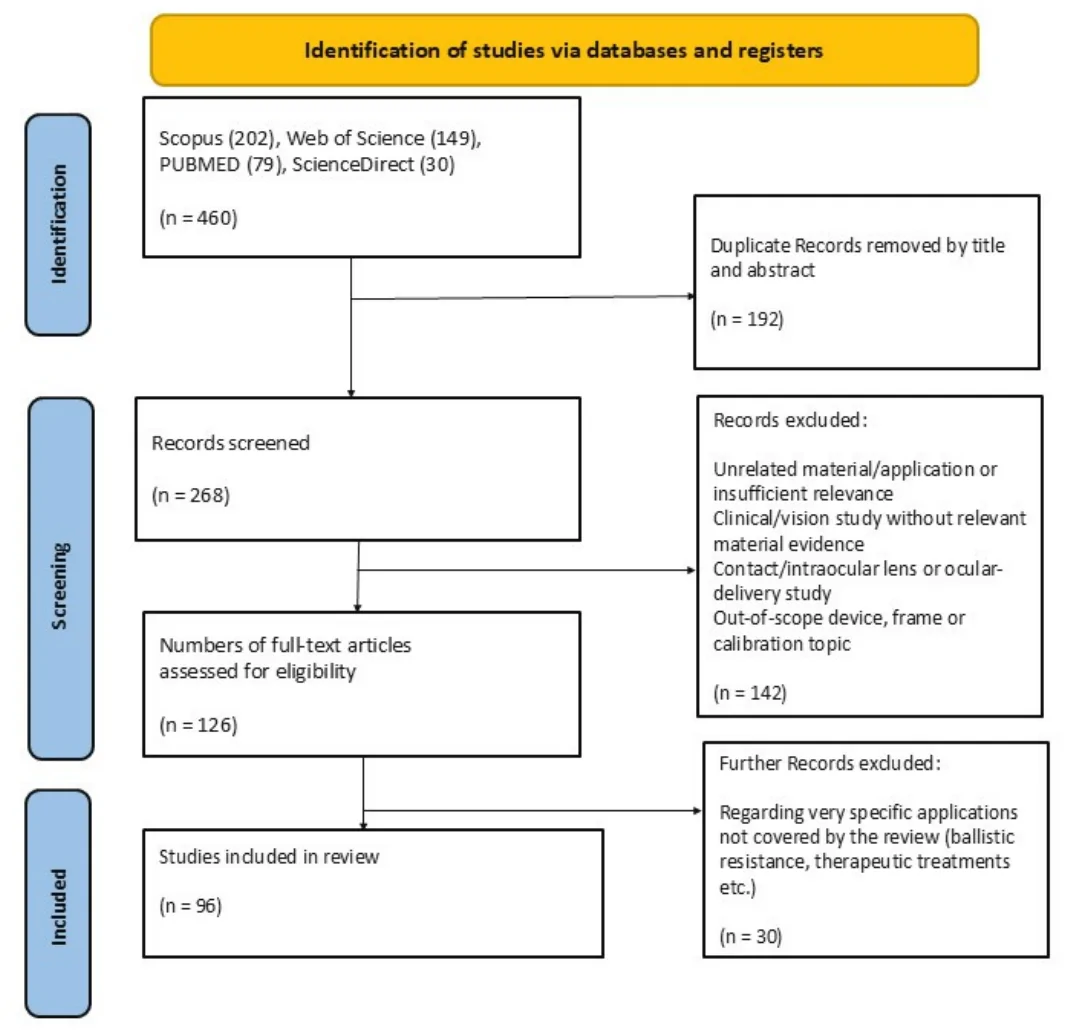
PRISMA 2020 flow diagram of the study selection process.

**Figure 2:**
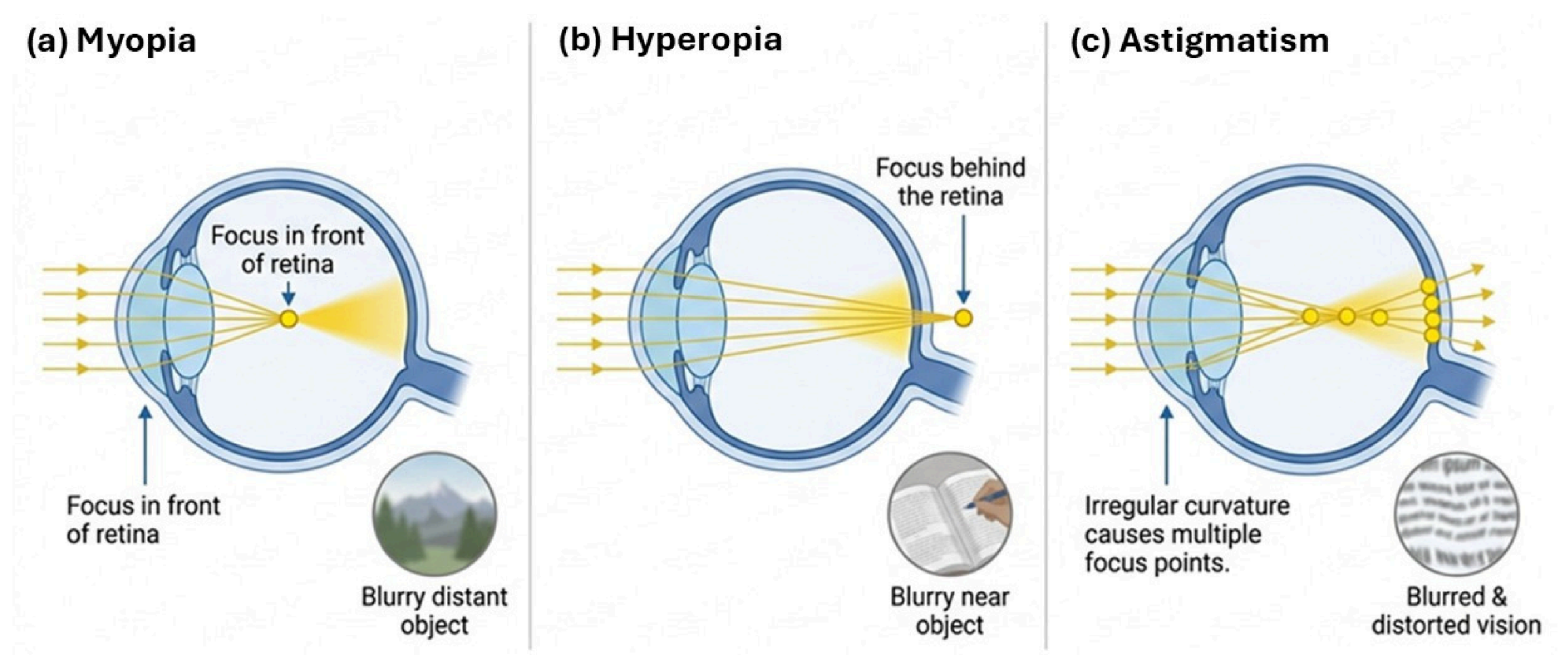
Common refractive errors of the human eye include (**a**) myopia (nearsightedness), where light rays focus in front of the retina, resulting in blurry vision for distant objects; (**b**) hyperopia (farsightedness), where light rays focus behind the retina, making close-up objects appear out of focus; and (**c**) astigmatism, where an irregular curvature of the cornea or lens creates multiple focal points, leading to blurred and distorted vision at all distances.

**Figure 3:**
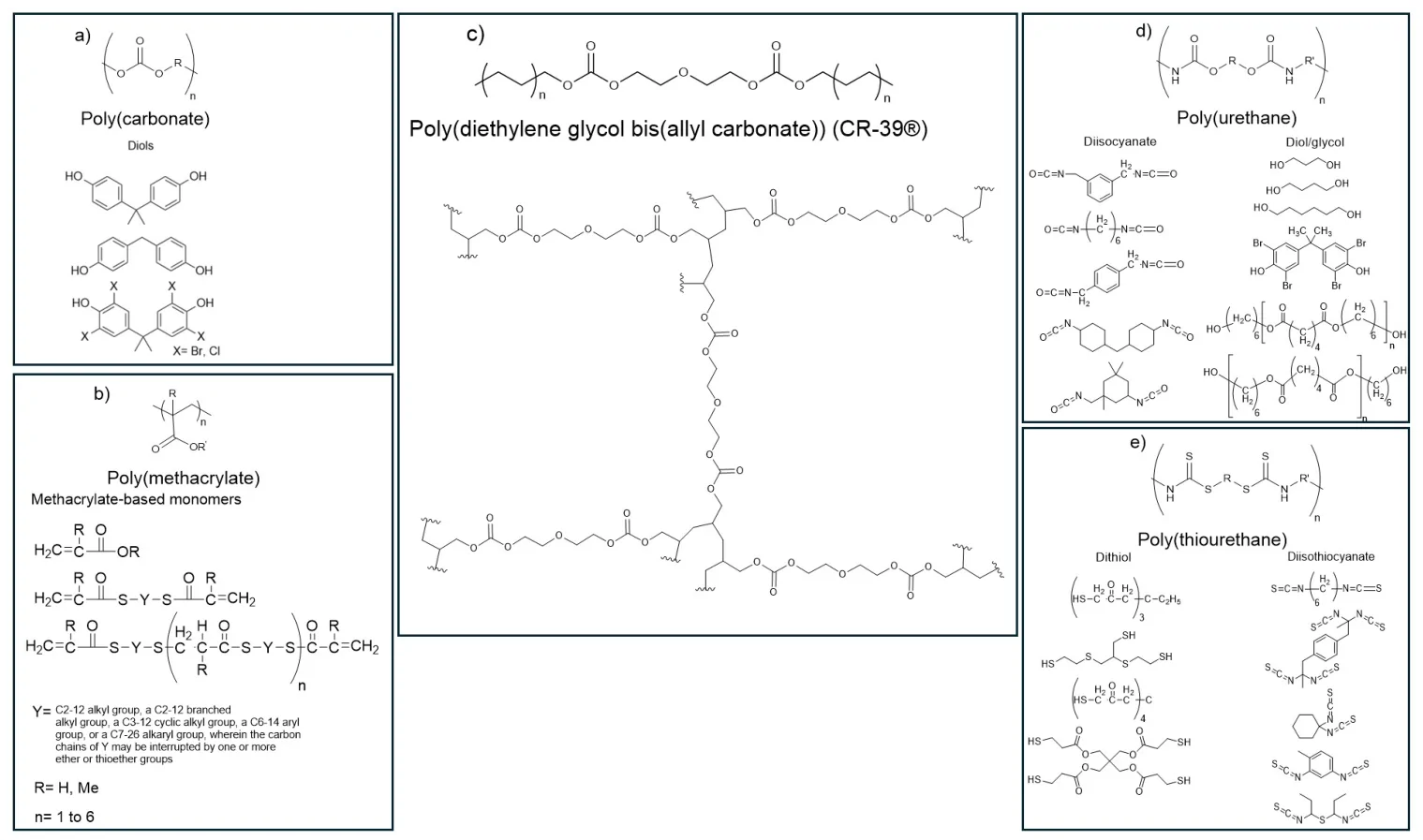
Main monomers and general polymer structure of (**a**) poly(carbonate) (PCs); (**b**) poly (methacrylate) (PMA); (**c**) poly(diethylene glycol bis(allyl carbonate)) (CR-39^®^); (**d**) poly(urethane); and (**e**) poly(thiourethane).

**Figure 4:**
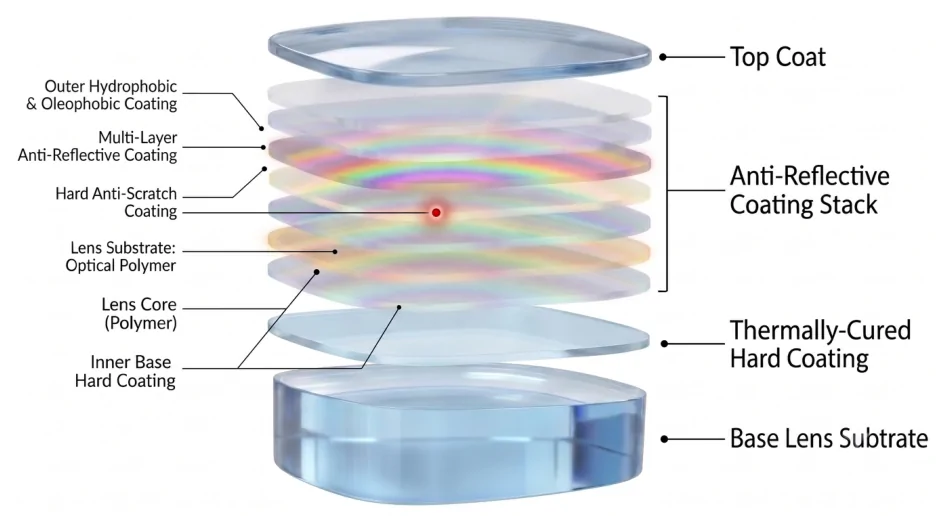
Schematic representation of modern lens coating.

**Table 1:** Ophthalmic lens main polymer families, architecture, processing techniques, and optical characteristics.

Polymer Family	Architecture	Processing	RI ^1^	Abbe Number	Density (g/cm^3^)	Main Advantages	Main Limitations
CR-39^® 2^	Crosslinked thermoset	Casting	1.50	59	1.3	Excellent optical quality	Low RI
BPA-PC ^3^	Linear thermoplastic	Injection moulding	1.59	31	1.2	High toughness	Surface hardness
PMMA ^4^	Linear thermoplastic	Injection moulding	1.49	58	1.2	Transparency	Low impact
Trivex^® 5^	Crosslinked thermoset	Casting	1.53	43–45	1.1	Balanced optics and toughness	Higher cost
MR^TM 6^	Crosslinked thermoset	Casting	1.60–1.74	32–42	1.6–1.8	High RI	Lower Abbe

^1^ RI: refractive index; ^2^ CR-39^®^: poly(diethylene glycol bis(allyl carbonate)); ^3^ BPA-PC: poly(bisphenol A carbonate); ^4^ PMMA: poly(methyl methacrylate); ^5^ Trivex^®^: poly(urethane-urea); ^6^ MR^TM^: poly(thiourethane).

**Table 2:** Coating types, chemical composition, deposition technology, and function.

Coating Type	Main Chemical Composition	Typical Deposition Method	Principal Function
Hard coating	Polysiloxanes, ORMOSILs, silica nanocomposites	Sol–gel, dip coating, spin coating	Scratch and abrasion resistance
Anti-reflective	SiO_2_, MgF_2_/TiO_2_, ZrO_2_, Ta_2_O_5_, Nb_2_O_5_, HfO_2_	Vacuum evaporation, sputtering, ion-assisted deposition	Reduce reflection, improve light transmission
Hydrophobic/ oleophobic	Fluorinated organosilanes, fluoropolymers	Vapor deposition, dip coating	Water and oil repellence
Mirror	Metallic (Cr, Ti, Al) or dielectric multilayers	PVD, sputtering	Reflective appearance, solar protection
Photochromic	Naphthopyrans, spirooxazines, spiropyrans	In-mass incorporation or surface treatment	Variable light transmission
Anti-fog	Hydrophilic polymers (e.g., PVA, polyurethane/ureas, polyacrylates), silica hybrids	Dip coating, plasma, sol–gel	Prevent fog formation

## Data Availability

No new data were created or analysed in this study. Data sharing is not applicable to this article.

## Supplementary Materials

The following supporting information can be downloaded at: https://www.mdpi.com/article/10.3390/molecules31203680/s1, Prisma 2020 checklist [97].

## Abbreviations

The following abbreviations are used in this manuscript:

BPA-PC	Poly(bisphenol A carbonate)
CR-39^®^	Poly(diethylene glycol bis(allyl carbonate))
PC	Polycarbonate
PMMA	Poly(methyl methacrylate)
PPG	Pittsburgh Plate Glass Industries
PRISMA	Preferred Reporting Items for Systematic Reviews and Meta-Analyses
RI	Refractive Index
UV	Ultraviolet
